# Supplementary material for: Harnessing gut microbes for glycan detection and quantification
Source: Nat Commun. 2023 Jan 17;14:275. doi: 10.1038/s41467-022-35626-2 (PMC9845299; doi:10.1038/s41467-022-35626-2)
Supplement: Supplementary file 1 — Supplementary Information [file 41467_2022_35626_MOESM1_ESM.pdf]

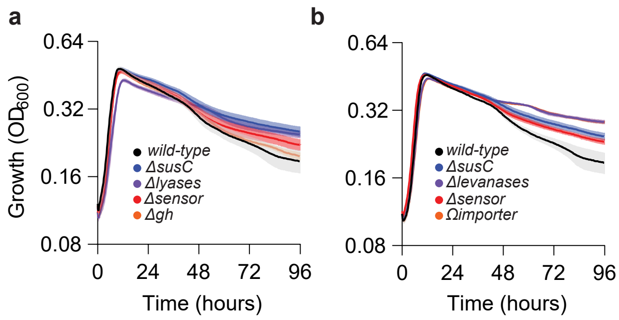

Supplementary Figure 1. PULs are not required for glycan-independent growth conditions. a Growth of *wild-type Bt* (black) or strains lacking a CS-inducible *susC* (*ΔBT3332,* blue), 3 CS-specific lyases (*ΔBT3324 ΔBT3350 ΩBT4410*, purple), CS-sensor (*ΔBT3334,* red), or the glucuronyl hydrolase (*ΔBT3348*, orange) were measured during anaerobic culture in minimal media containing galactose as a sole carbon source. **b** Growth of *wild-type Bt* (black) or strains lacking a levan-inducible *susC* (*ΔBT1763*, blue), 4 levanases (*Δ BT1760-1759 ΔBT3082 ΩBT1765*, purple), fructan-sensor (*ΔBT1754,* red), or a putative inner membrane fructose importer (*ΩBT1758,* orange) were measured during anaerobic culture in minimal media containing galactose as a sole carbon source. Values are the mean of 8 biological replicates and error bars are SEM in color-matched shading. Source data are provided as a Source Data file.

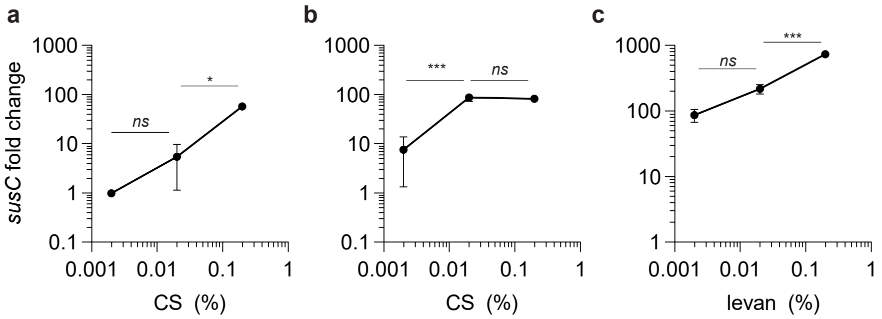

Supplementary Figure 2. PULs exhibit dose-dependent transcriptional responses. a-c The fold increase of (**a&b**) *BT3332* or (**c**) *BT1763* mRNA levels in *wild-type Bt* following the introduction of mixtures containing either 0.2%, 0.02%, or 0.002% (**a&b**) CS or (**c**) levan supplemented with galactose to 0.5% total carbohydrate. The fold increase was calculated as the change in transcript levels between cultures before and after (**a**) 2 hours or (**b&c**) 1 hour following induction of glycan mixtures. Values are the mean of 6 independent measurements, error bars represent SEM, and P-values were calculated by 2-way ANOVA with Tukey's honest significance test and *** represents values < 0.001, * < 0.05, and *ns* indicates values > 0.05. Source data are provided as a Source Data file.

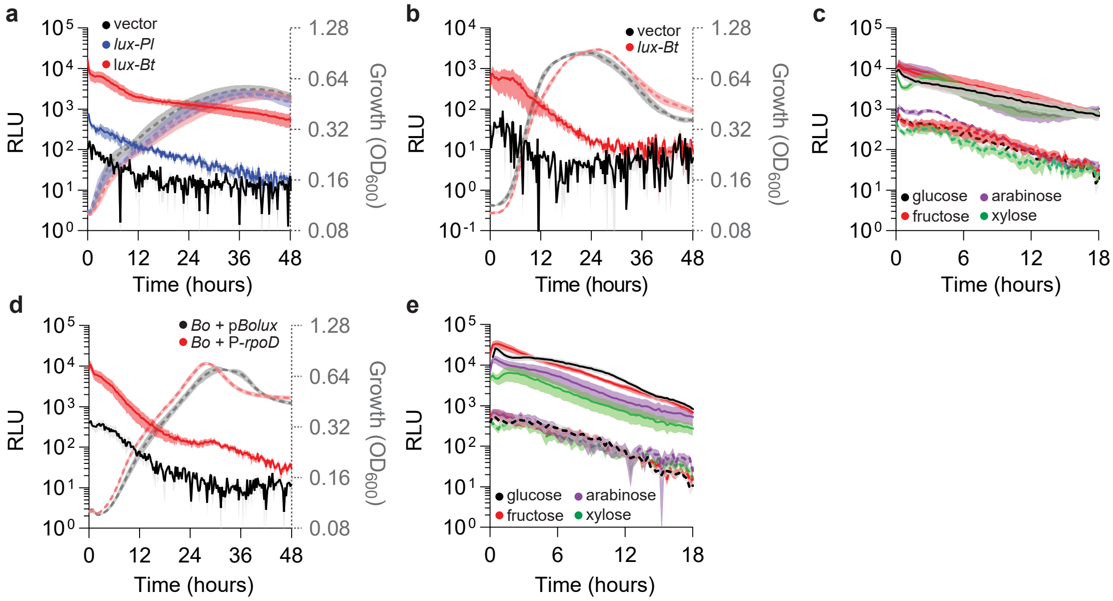

Supplementary Figure 3. Bioluminescence during anaerobic growth across *Bacteroides* species and growth conditions. **a** Relative luminescence (solid lines) or growth (dashed lines) from *Bt* strains harboring an empty multi-copy vector (black) or plasmids containing either the *lux* operon from *Pl* (*lux-Pl*, blue) or the Bacteroides-optimized *lux* cassette (lux-*Bt*, red) expressed from the *Bt rpoD* promoter and rpiL* RBS was measured during growth in minimal media containing glucose as the sole carbon source. **b** Relative luminescenece from *wild-type Bt* strains harboring a single-copy empty vector (black) or a plasmid encoding the Bacteroides optimized *lux* cassette (red) grown in galactose as the sole carbon source. **c** The relative luminescence of *Bt* strains harboring p*Bolux* (dashed lines) or a plasmid with the corresponding *rpoD* promoter cloned into the BamHI and SpeI sites (solid lines) during growth in glucose (black), fructose (red), arabinose (purple), or xylose (green) as the sole carbon source. **d** The relative luminescence (solid lines) or growth (dashed lines) of *Bo* strains harboring p*Bolux* (black) or a plasmid with the *Bo rpoD* promoter cloned into the BamHI and SpeI sites (red) during growth in galactose as the sole carbon source. **e** The relative luminescence of *Bo* strains described in panel **d** during growth in glucose (black), fructose (red), arabinose (purple), or xylose (green) as the sole carbon source. For all panels, values are the mean of 8 biological replicates and error is SEM in color-matched shading. Source data are provided as a Source Data file.

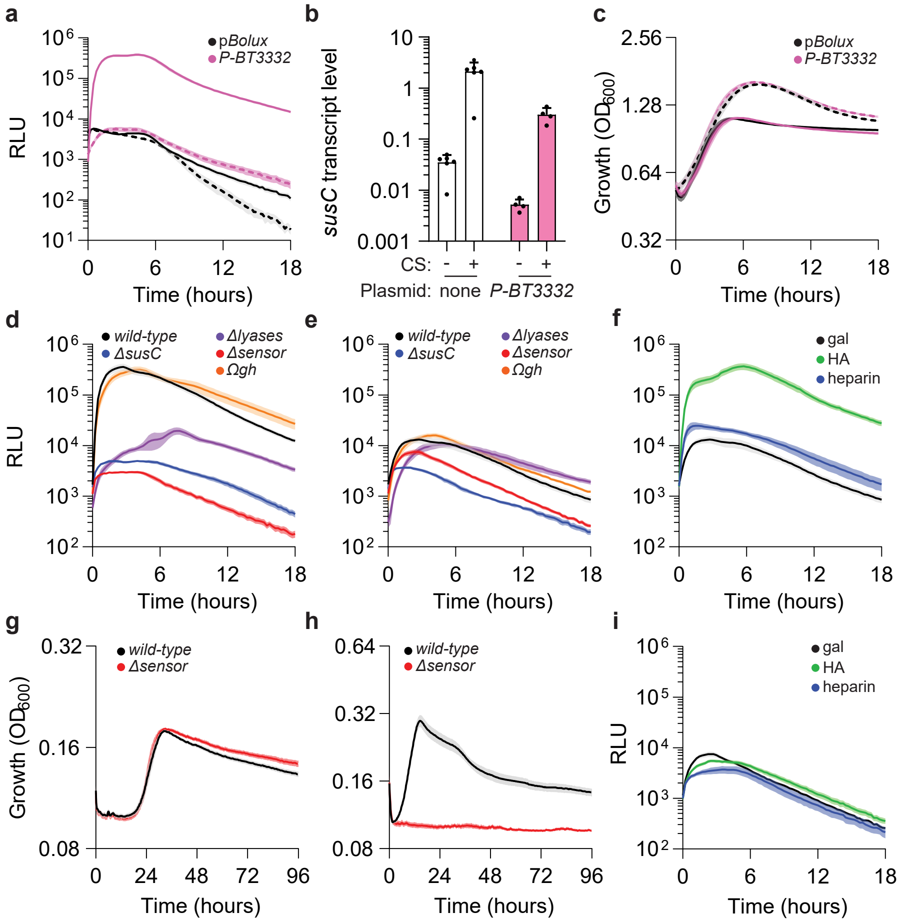

Supplementary Figure 4. The *BT3332* promoter confers CS and HA-inducible bioluminescence in p*Bolux*. a Relative luminescence of *wild-type Bt* harboring p*Bolux* (black) or a plasmid including the promoter region preceding the CS-inducible *susC* (P-*BT3332*, pink) following introduction of CS (solid lines) or galactose (dashed lines) as the sole carbon source. **b** *susC* transcript levels (*BT3332*) in *wild-type Bt* strains without a plasmid (open bars) or harboring *P-BT3332* (pink bars) grown in galactose or a mixture of galactose and CS. Values are the mean of at least 4 measurements and error bars are standard deviation. **c** Growth of strains described in (**a**) following introduction of CS (solid lines) or galactose (dashed lines) as the sole carbon source. For panels **a&c**, values are the mean of 12 biological replicates and error is SEM in color-matched shading. **d&e** Relative luminescence from *wild-type Bt* (black) or strains lacking the CS-inducible *susC* (*ΔBT3332*, blue), 3 CS-specific lyases (*ΔBT3324 ΔBT3350 ΩBT4410*, purple), the CS-sensor (*ΔBT3334,* red), or the glucuronyl hydrolase (*ΔBT3348*, orange) harboring P-*BT3332* following the introduction of an equal mixture of (**d**) CS and galactose or (**e**) galactose alone. **f** Relative luminescence from *wild-type Bt* harboring P-*BT3332* following the introduction of galactose alone (black) or an equal mixture of galactose and HA (green) or heparin (blue). **g&h** Growth of *wild-type Bt* (black) or a strain lacking the CS-sensor (*ΔBT3334,* red) were measured during anaerobic culture in minimal media containing (**g**) heparin or (**h**) HA as a sole carbon source. **i** Relative luminescence from a CS-sensor deficient *Bt* strain (*ΔBT3334*) harboring P-*BT3332* following the introduction of galactose alone (black) or an equal mixture of galactose and HA (green) or heparin (blue). For panels **d**-**i**, values are the mean of 8 biological replicates and error bars are SEM in color-matched shading. Source data are provided as a Source Data file.

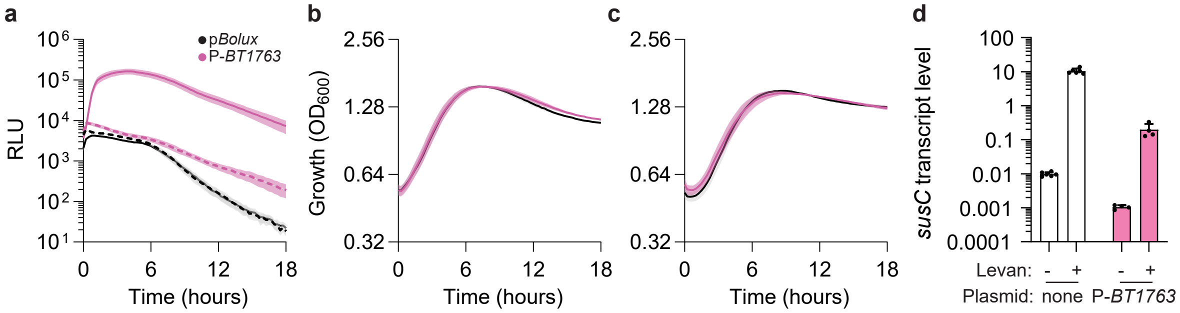

Supplementary Figure 5. A fructan-responsive PUL reporter exhibits levan-inducible activity in *Bt*. a Relative luminescence from *wild-type Bt* harboring p*Bolux* (black) or a plasmid including the promoter region upstream of the levan-inducible *susC* (P-*BT1763*, pink) following the introduction of levan (solid lines) or galactose (dashed lines) as the sole carbon source. **b&c** Growth of strains described in (**a**) following the introduction of (**b**) galactose or (**c**) levan as the sole carbon source. For panels **a-c**, values are the mean of 12 biological replicates and error is SEM in color-matched shading. **d** *susC* transcript levels (*BT1763*) in *wild-type Bt* strains without a plasmid (open bars) or harboring P-*BT1763* (pink bars) grown in galactose or a mixture of galactose and levan. Values are the mean of 4 (P-*BT1763*) or 6 (no plasmid) measurements and error bars are standard deviation. Source data are provided as a Source Data file.

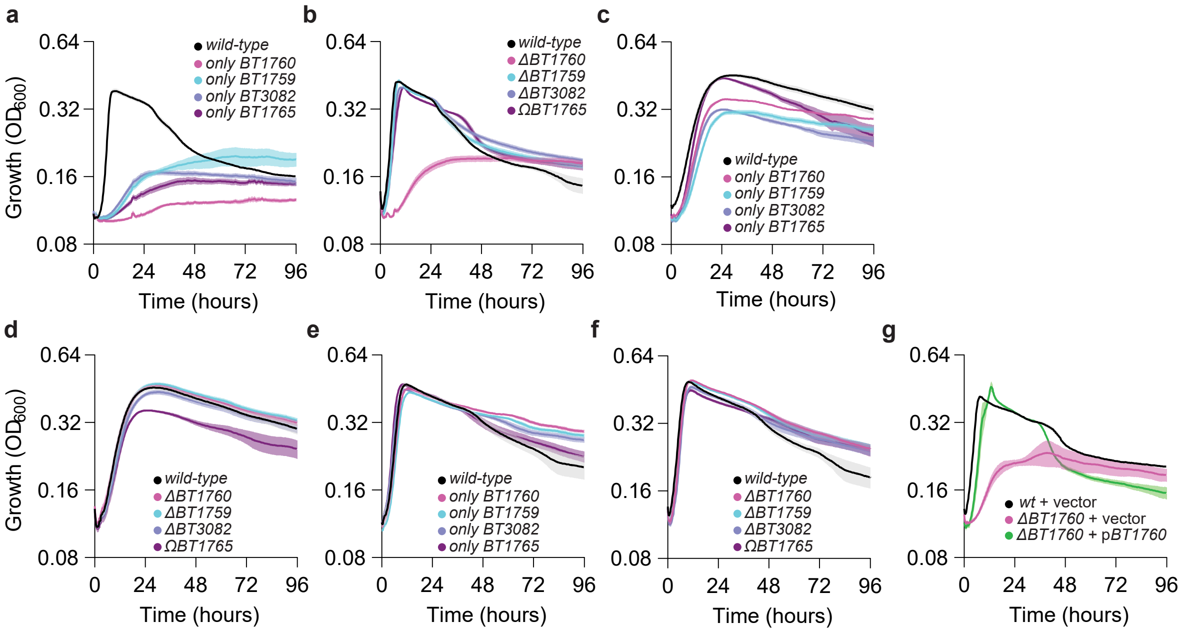

Supplementary Figure 6. Multiple levanases coordinate *Bt* fructan utilization. a Growth of *wild-type Bt* or strains lacking all other levanases except *BT1760* (*ΔBT1759 ΔBT3082 ΩBT1765,* pink), *BT1759* (*ΔBT1760 ΔBT3082 ΩBT1765*, teal), *BT3082* (*Δ BT1760-59 ΩBT1765,* lavender), or *BT1765* (*ΔBT1760-59 ΔBT3082,* purple) in 0.1% levan as a sole carbon source. **b** Growth of *wild-type Bt* (black) or strains lacking *BT1760* (pink), *BT1759* (teal), *BT3082* (lavender), or *BT1765* (purple) in 0.1% levan as a sole carbon source. **c&e** Growth of strains described in panel **a** cultured in either 0.1% (**c**) fructose or (**e**) galactose as a sole carbon source. **d&f** Growth of strains described in panel **b** cultured in either 0.1% (**d**) fructose or (**f**) galactose as a sole carbon source. **g** Growth of *wild-type Bt* (black) or *BT1760*-deficient strains (pink) harboring empty pNBU2 or a plasmid encoding *BT1760* (green) in 0.1% levan as the sole carbon source. Values are the mean of 8 biological replicates and error is SEM in color-matched shading. Source data are provided as a Source Data file.

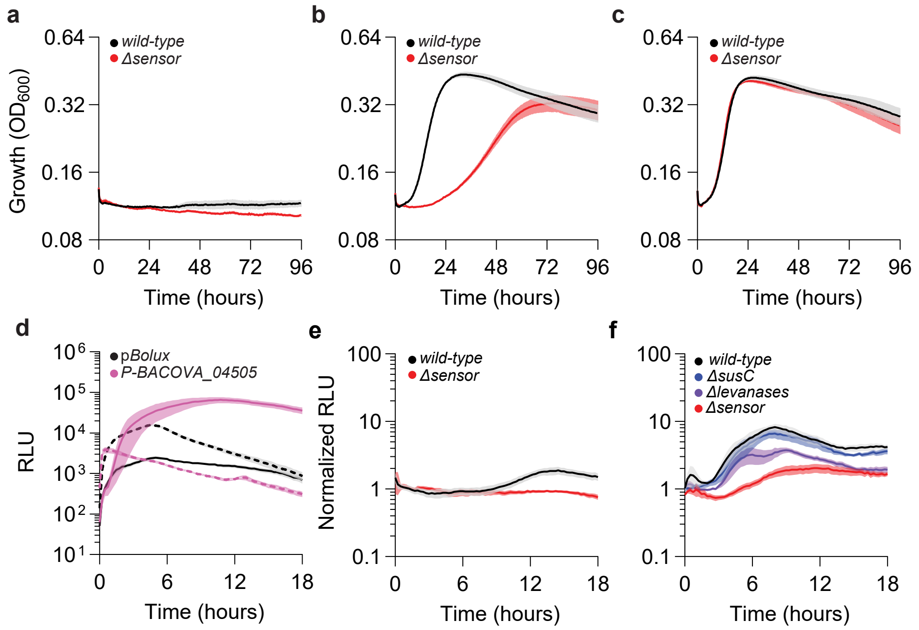

Supplementary Figure 7. A fructan-responsive PUL reporter exhibits inulin-inducible activity in *Bo*. a-c Growth of *wild-type Bo* (black) or a strain lacking the *Bo* inulin sensor (*ΔBACOVA_04496*, red) were measured during anaerobic culture in minimal media containing (**a**) levan, (**b**) fructose, or (**c**) galactose as sole carbon sources. **d** Relative luminescence from *wild-type Bo* harboring p*Bolux* (black) or a plasmid that includes the promoter region preceding the inulin-inducible *susC* (P-*BACOVA_04505*; pink) were measured following the introduction of inulin (solid lines) or galactose (dashed lines) as the sole carbon source. Values are the mean of 12 biological replicates and error is SEM in color-matched shading. **e** Relative luminescence from *wild-type Bo* harboring P-*BACOVA_04505* (black) or an isogenic strain lacking the *Bo* inulin sensor (*ΔBACOVA_04496*, red) was measured following the introduction of an equal mixture of galactose and levan and subsequently normalized by the relative luminescence from identical cultures supplied galactose alone. **f** Relative luminescence from *wild-type Bt* (black) or strains lacking the levan-inducible *susC* (*ΔBT1763*, blue), 4 levan-specific hydrolases (*Δ BT1760-1759 ΔBT3082 ΩBT1765*, purple) or the *Bt* fructan sensor (*ΔBT1754,*red) harboring P-*BT1763* were measured following the introduction of an equal mixture of inulin and galactose normalized by the relative luminescence of identical cultures supplied galactose alone. For panels **a-c** and **e&f**, values are the mean of 8 biological replicates and error is SEM in color-matched shading. Source data are provided as a Source Data file.

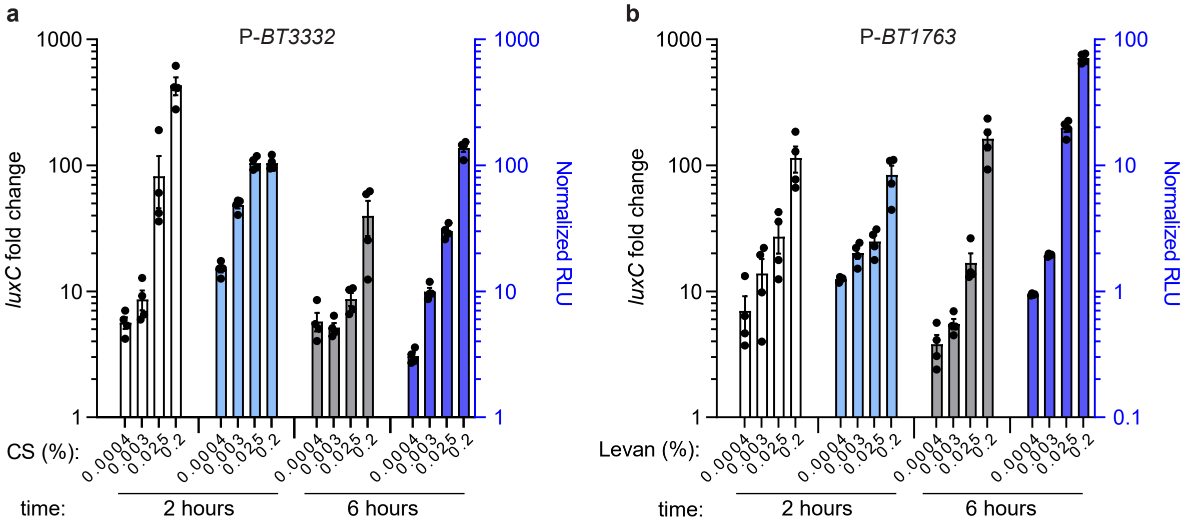

Supplementary Figure 8. PUL reporters confer dose-dependent *lux* transcription and activity. a&b The fold increase in *luxC* transcript levels (open and gray bars, left Y-axis) and corresponding luminescence (blue bars, right Y-axis) from *wild-type Bt* strains harboring (**a**) P-*BT3332* or (**b**) P-*BT1763* after 2 and 6 hours following the introduction of decreasing concentrations of either (**a**) CS or (**b**) levan containing galactose to a total carbohydrate content of 0.5% and normalized by identical cultures supplied galactose alone. Values are the mean of 4 independent measurements and error bars are SEM. Source data are provided as a Source Data file.

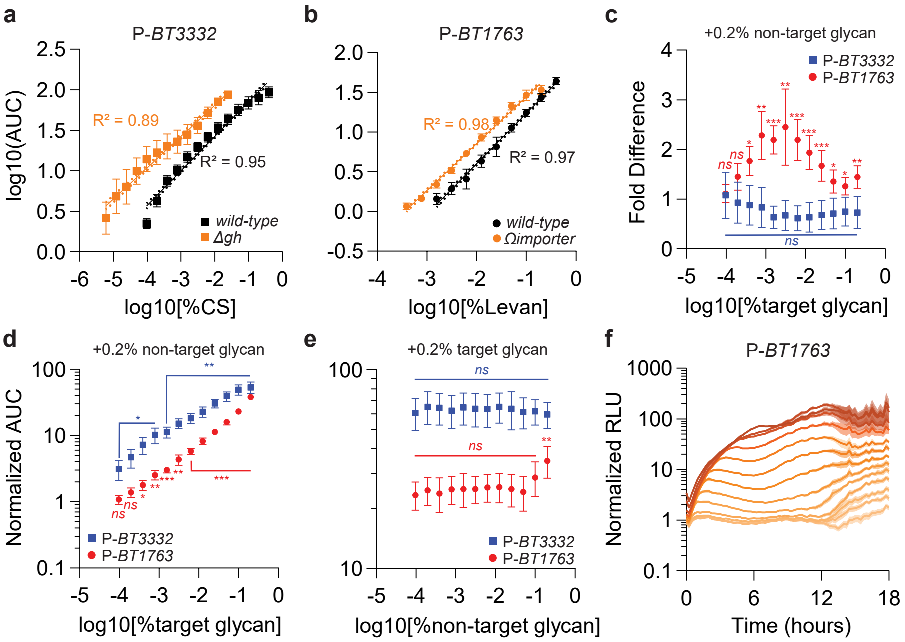

Supplementary Figure 9. PUL reporters display concentration dependent responses to target glycans. a&b The log10 AUC of significant responses from *wild-type Bt* (black) or mutants defective for PUL-sensor deactivation (orange) harboring (**a**) P-*BT3332* or (**b**) P-*BT1763* and supplied 2-fold serial dilutions of 0.4% (**a**) CS or (**b**) levan with galactose to a total carbohydrate content of 0.5% and normalized to responses from identical cultures supplied galactose alone. Values are the mean of 12 biological replicates and error is standard deviation. Solid lines represent the simple linear regression models corresponding to responses from each strain and color-matched dashed lines represents the 95% confidence intervals. **c** The fold difference between the AUC of responses from *wild-type Bt* strains harboring either P-*BT3332* (blue squares) or P-*BT1763* (red circles) supplied glycan mixtures containing 2-fold serial dilutions of 0.2% CS or levan, respectively, in the presence or absence of constant 0.2% levan or CS, respectively, and containing galactose to 0.5% total carbohydrate normalized by the AUC of responses from identical cultures supplied galactose alone. **d** The AUC of responses from *wild-type Bt* strains harboring either P-*BT3332* (blue squares) or P-*BT1763* (red circles) supplied glycan mixtures containing 2-fold serial dilutions of 0.2% CS or levan, respectively, in the presence of constant 0.2% levan or CS, respectively, and containing galactose to 0.5% total carbohydrate normalized by the AUC of responses from identical cultures supplied galactose alone. **e** The AUC of response curves measured from *wild-type Bt* harboring P-*BT3332* (blue squares) supplied a mixture containing 2-fold serial dilutions of levan, constant 0.2% CS and with galactose to a total 0.5% carbohydrate content and normalized by identical cultures supplied galactose alone. The AUC of responses from *wild-type Bt* harboring a levan-responsive reporter (red circles) supplied a mixture containing 2-fold serial dilutions of CS, constant 0.2% levan and with galactose to a total 0.5% carbohydrate content normalized by identical cultures supplied galactose alone. For panels **c-e**, values represent the mean of 6 biological replicates, error bars are standard deviation, and P-values were calculated using 2-way ANOVA with Tukey's honest significance test and *** represents values < 0.001, ** < 0.01, * < 0.05, and *ns* > 0.05. **f** Relative luminescence from a *BT1758*-deficient *Bt* strain harboring P-*BT1763* following the introduction of 2-fold serial dilutions of 0.4% levan containing galactose to a total carbohydrate content of 0.5% and normalized to identical cultures supplied galactose alone. Values are the mean of 12 biological replicates and error bars are SEM in color-matched shading. Source data are provided as a Source Data file.

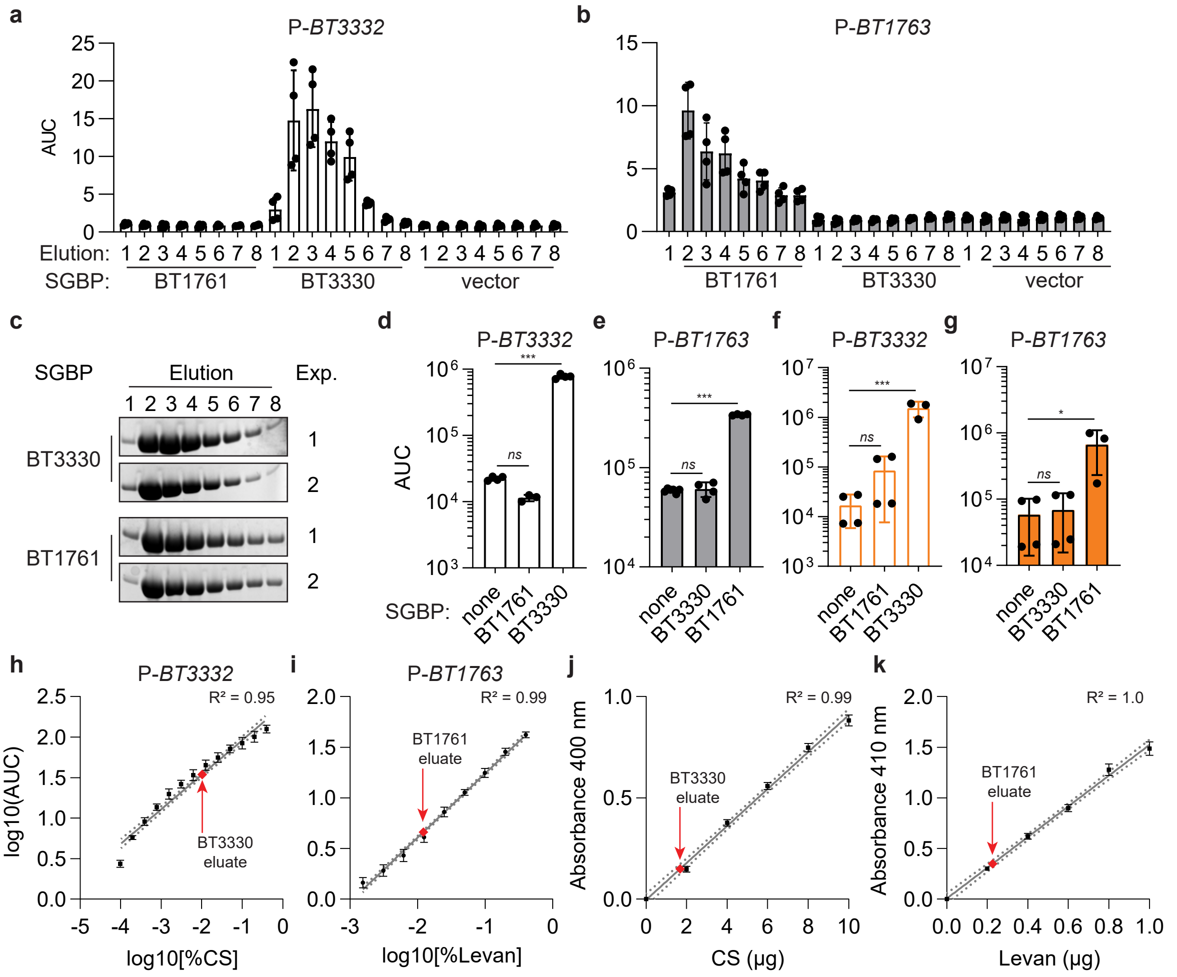

Supplementary Figure 10. PUL reporters can indicate unknown target glycan abundances. a&b The AUC of responses from *wild-type Bt* strains harboring either (**a**) P-*BT3332* (open bars) or (**b**) P-*BT1763* (filled bars) supplied elutions from nickel-NTA agarose incubated with *E. coli* whole cell lysates from strains containing empty pT7-7 vector, or plasmids engineered to over-express BT1761 or BT3330 and pre-incubated with a mixture of 0.1% levan and CS. All elutions were supplemented with 0.2% galactose. Values represent a mean of 4 total replicates from two independent experiments and error bars are SEM. **c** Coomassie stained SDS-PAGE gels showing the corresponding protein levels for BT3330 (top 2 gels) or BT1761 (bottom 2 gels) in each elution fraction. **d&e** The AUC of responses from *wild-type Bt* strains harboring either (**d**) P-*BT3332* (open bars) or (**e**) P-*BT1763* (filled bars) supplied galactose alone or concentrated material co-purifying with BT3330 or BT1761 supplemented with 0.4% galactose. For panel **d**, values are the mean of 3 (BT1761 eluate) or 4 (no SGBP and BT3330 eluates) measurements. For panel **e**, values are the mean of 4 measurements. **f&g** The AUC of responses from deactivation defective *Bt* strains harboring either (**f**) P-*BT3332* (open bars) or (**g**) P-*BT1763* (filled bars) supplied galactose alone or concentrated material co-purifying with BT3330 or BT1761 supplemented with 0.4% galactose. For panel **f**, values are the mean of 3 (BT3330 eluate) or 4 (no SGBP and BT1761 eluates) measurements. For panel **g**, values are the mean of 3 (BT1761 eluate) or 4 (no SGBP and BT3330 eluates) measurements. For panels **d-g**, error is standard deviation and P-values were computed using 1-way ANOVA with Tukey's honest significance test and *** represents values < 0.001, * < 0.05, and *ns* indicates values > 0.05. **h&i** The AUC from *wild-type Bt* strains harboring either (**h**) P-*BT3332* or (**i**) P-*BT1763* supplied pooled, concentrated elutions in combination with 0.4% galactose and normalized to identical cultures supplied galactose alone. Measurements were collected alongside identical strains supplied 2-fold serial dilutions of either (**h**) CS or (**i**) levan with galactose to a total carbohydrate content of 0.5% and normalized to identical cultures supplied galactose alone. Linear regression models (gray line) were computed in Prism, and sample concentrations (red) were estimated with the derived equations. Values are the mean of 4 technical replicates, error bars are standard deviation, and the dashed gray lines are the 95% confidence interval. **j&k** The estimated (**j**) total glycosaminoglycan or (**k**) fructan content of samples described for panels **h** and **i**, respectively, using colorimetric glycan assay kits. Values are the mean of 3 technical replicates, error bars are standard deviation, and the dashed gray lines are the 95% confidence interval. Source data are provided as a Source Data file.

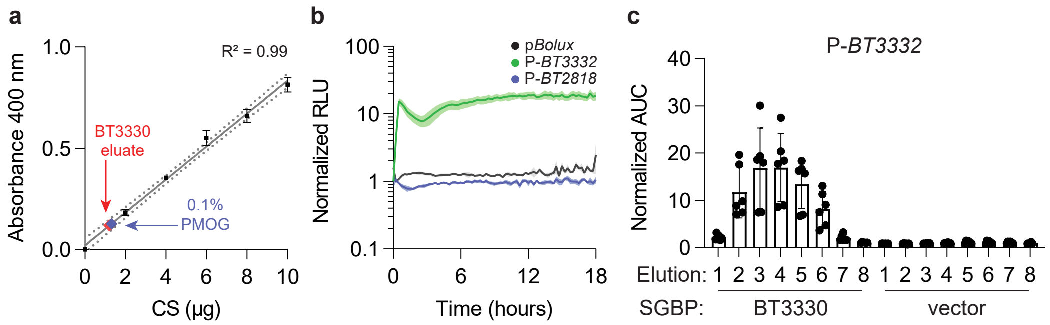

Supplementary Figure 11. Detection and isolation of target glycans from biologically-derived mixtures. a The estimated total glycosaminoglycan content in a 0.1% PMOG solution (blue) or material co-purifying with BT3330 pre-incubated with PMOG (red) were measured using a colorimetric assay against CS standards. Values are the mean of 2 technical replicates, error bars are standard deviation, and the dashed gray lines are the 95% confidence intervals. **b** Relative luminescence from *wild-type Bt* strains harboring p*Bolux* (black), P-*BT3332* (green), or P-*BT2818* (blue) following the addition of a mixture of galactose and CS di0S normalized by responses from identical strains supplied galactose alone. Values are the mean of 4 biological replicates and error is SEM in color matched shading. **c** The AUC of responses from *wild-type Bt* strains harboring P-*BT3332* supplied elution fractions from nickel-NTA agarose combined with *E. coli* whole cell lysates from strains containing empty pT7-7 vector or a plasmid engineered to over-express BT3330 and pre-incubated with a 0.1% PMOG solution. All elutions fractions were supplemented with 0.2% galactose. Values represent the mean of 6 measurements from 3 independent experiments and error bars are standard deviation. Source data are provided as a Source Data file.

Supplementary Table 1. Strains used in this Study.

Name	Genotype	Plasmid	Reference
E. coli			
S17-1	*λpir*		[1]
BL21 (DE3)	*E. coli B dcm ompT hsdS(rB-mB-) gal λDE3*		[2]
B. thetaiotaomicron (VPI-5482)			
GT23	*Δtdk*		[3]
VR69	*Δtdk ΔBT3348*		[4]
GT150	*Δtdk ΔBT3334*		[4]
GT165	*Δtdk ΔBT1754*		this work
GT962	*Δtdk att-1::pNBU2-lux-Pl*		this work
GT1039	*Δtdk att-1::pNBU2-tetQ*		this work
GT1059	*Δtdk att-1::pNBU2-lux-Bt*		this work
GT3137	*Δtdk*	p-*lux-Pl*	this work
GT1541	*Δtdk*	p-*lux-Bt*	this work
GT1866	*Δtdk*	pLYL01	this work
GT1867	*Δtdk*	p*Bolux*	this work
GT1868	*Δtdk*	P-*Bt-rpoD*	this work
GT1893	*Δtdk*	P-*BT1763*	this work
GT1934	*Δtdk*	P-*BT3332*	this work
GT2111	*Δtdk att-1::pNBU2-ermG*		this work
GT2618	*Δtdk ΔBT3334*	P-*BT3332*	this work
GT2620	*Δtdk ΔBT1754*	P-*BT1763*	this work
GT2926	*Δtdk ΔBT3332*		this work
GT2939	*Δtdk ΔBT3332*	P-*BT3332*	this work
GT3086	*Δtdk ΔBT3324 ΔBT3350 BT4410::pKNOCK-ermGb*		this work
GT3102	*Δtdk ΔBT3348*	P-*BT3332*	this work
GT3117	*Δtdk ΔBT3324 ΔBT3350 BT4410::pKNOCK-ermGb*	P-*BT3332*	this work
GT3181	*Δtdk ΔBT1760*		this work
GT3192	*Δtdk ΔBT1760*	P-*BT1763*	this work
GT3196	*Δtdk ΔBT1763*		this work
GT3199	*Δtdk ΔBT1763*	P-*BT1763*	this work
GT3215	*Δtdk ΔBT1760 att-1::pNBU2-ermG*		this work
GT3216	*Δtdk ΔBT1760 att-1::pNBU2-ermG-*P_{BT1763}*-BT1760*		this work
GT3226	*Δtdk ΔBT1759*		this work
GT3246	*Δtdk ΔBT1759*	P-*BT1763*	this work
GT3282	*Δtdk BT1765::pKNOCK-ermG*		this work
GT3299	*Δtdk BT1765::pKNOCK-ermG*	P-*BT1763*	this work
GT3303	*Δtdk ΔBT3082*		this work
GT3308	*Δtdk ΔBT1760-59 ΔBT3082*		this work
GT3346	*Δtdk ΔBT1760 ΔBT3082 BT1765::pKNOCK-ermGb*		this work
GT3347	*Δtdk ΔBT1759 ΔBT3082 BT1765::pKNOCK-ermGb*		this work
GT3348	*Δtdk ΔBT1760-59 ΔBT3082 BT1765::pKNOCK-ermG*		this work
GT3358	*Δtdk ΔBT1759 ΔBT3082 BT1765::pKNOCK-ermGb*	P-*BT1763*	this work
GT3356	*Δtdk ΔBT1760 ΔBT3082 BT1765::pKNOCK-ermGb*	P-*BT1763*	this work
GT3360	*Δtdk ΔBT1760-59 ΔBT3082 BT1765::pKNOCK-ermG*	P-*BT1763*	this work
GT3379	*Δtdk BT1758:pKNOCK-ermG*		this work
GT3393	*Δtdk BT1758:pKNOCK-ermG*	P-*BT1763*	this work
GT3534	*Δtdk ΔBT3082*	P-*BT1763*	this work

GT1912	Δ*tdk*	P-*BT2818*	this work
GT2813	Δ*tdk ΔBT2826*	P-*BT2818*	this work
GT2917	Δ*tdk ΔBT3334*	P-*BT2818*	this work
GT4618	Δ*tdk ΔBT2826*	P-*BT3332*	this work
B. ovatus (ATCC 8483)			
ATCC 8483			ATCC
GT3173		P-*BACOVA_04505*	this work
GT3179	*BACOVA_04495*::pSIE1-*ΔBACOVA_04496*		this work
GT3183	*ΔBACOVA_04496*		this work
GT3189	*ΔBACOVA_04496*	p*Bolux*	this work
GT3190	*ΔBACOVA_04496*	P-*BACOVA_04505*	this work
GT3489		p*Bolux*	this work
GT3490		P-*Bo-rpoD*	this work

Supplementary Table 2. Primers Used in this Study.

identifier	name	sequence (5' → 3')	purpose
**qPCR**			
1044	qBT16s_f	ggtagtccacacagtaaacgatgaa	measure 16s rRNA levels using qPCR
1045	qBT16s_r	cccgtcaaattcctttgagtttc	
1060	qBT3332_f	tggttgtcggctatcaggaagt	measure *BT3332* mRNA levels using qPCR
1061	qBT3332_r	acatctgccatgttggctttc	
1056	qBT1763_f	agcgtaaagccgacctgaca	measure *BT1763* mRNA levels using qPCR
1057	qBT1763_r	tcaccttgcttctggatttcg	
2208	qluxC_f	tgcgccatcttatgctgatg	measure *luxC* mRNA levels using qPCR
2209	qluxC_r	tgcggacgtcaaatcaacag	
***lux* reporter construction**			
w2952	pNBU-P-BT1311_f	gctctagaactagtggatcctgatctggaagaagcaatgaaagct	clone the *Bt rpoD* promoter preceding the rpiL* RBS
w2905	rpiL*_r	catattcgtttaattaaataaataatttattttattttaaa	
w3115	rpiL*-luxC_f	atttatttaattaaacgaatatgactaaaaaaatttcattcattattaacgg	clone the *P. luminescens lux* cassette preceded by the rpiL* RBS into pNBU2
w3124	luxE-nbu_r	aagataggcaattagtcgactcaactattaaatgcttggtttaagcttaa	
w3265	luxC_r	tttacaatttgccatgcggattacgggacaaatacaaggaacttatc	clone the *Bacteroides*-optimized *lux* cassette preceded by the rpiL* RBS into pNBU2
w3266	luxD_f	tccgcatggcaaattgtaaattgtaaatcgtaaaatagtaatatattaatggaaaataaatccaaatataaaaccatc	
w3267	luxD_r	attctttatcctcctccttattaagacagcgaaatcgcttga	
w3268	luxE_f	taaggaggaggataaagaatatgacttcatatgttgataaacaagagatc	
w3269	luxE_r	agtgttaccttcattcatccttcttcacccttcatttatatcaactattaaatgcttggtttaagctt	
w3270	luxA_f	ggatgaatgaaggtaacactcataaactcgaaattcttcattcttaatttttaattaaaatatatgaaatttggaaactttttgcttac	
w3271	luxA_r	ccattgtcttattcctttctttataatagcgaacgttgtttttctttaag	

w3272	luxB_f	agaaaggaataagacaatggatatgaaatttggattgttcttccttaac	
w3273	luxB_r	aagataggcaattagtcgacttacatgtggtactttttaatattatcatcaacaa	
1080	pLYL-SpeI-rpil*_f	gctcggtacccggggatccactagtcactcccgcattttaaaataaaataaattatttatttaattaaacg	clone the *Bacteroides*-optimized *lux* cassette into pLYL01 and introduce a SpeI site
1011	pLYL-luxB_r	tgcatgcctgcaggtcgacttacatgtggtactttttaatattatcatcaacaatattg	
1081	pBolux-P-BT1311_f	gctcggtacccggggatcctgatctggaagaagcaatgaaagct	clone the *Bt rpoD* promoter into p*Bolux*
1082	pBolux-P-BT1311_r	aaatgcgggagtgactagtcaaagttacgacaaataatttgttaacatacatatttaggc	
2109	pBolux-Bo-PrpoD_f	gctcggtacccggggatccatctggaagaagtaatgaaagctgc	clone the *Bo rpoD* promoter into p*Bolux*
2110	pBolux-Bo-PrpoD_r	aaatgcgggagtgactagtcaaagttacgacaaataatttgttaacatacaaaa	
1150	pBolux-pBT1763_f	gctcggtacccggggatcctatcattcagttttctgttggttactttgagtga	clone the *BT1763* promoter into p*Bolux*
1304	pBolux-pBT1763_r	aaatgcgggagtgactagttagtttaatgttattaatttaaaagtacgaattttctcttttcgatg	
1232	pBolux-pBT3332_f	gctcggtacccggggatccaaaatggaactgggcaatgacagg	clone the *BT3332* promoter into p*Bolux*
1373	pBolux-pBT3332_r	aaatgcgggagtgactagtcctttttcttgtctggttggatagatgtttttt	
1943	pBolux-pBACOVA_04505_f	gctcggtacccggggatccttgtttgttggagattgttttcatatcgttg	clone the *BACOVA_04505* promoter into p*Bolux*
1944	pBolux-pBACOVA_04505_r	aaatgcgggagtgactagttagtttgatgttattaaattaaaagtacgaattttac	
1188	pBolux-pBT2818_f	gctcggtacccggggatcctgttccttacaaagcctccttttcca	clone the *BT2818* promoter into p*Bolux*
1351	pBolux-pBT2818_r	aaatgcgggagtgactagtactgtttaatttgataggttaataatatatttagtttatagttaggtcaac	
**Engineering mutations**			
1846	pEXC-ΔBT3332_5f	gctctagaactagtggatccgacagcctccagctgacgg	engineer a chromosomal deletion of *BT3332*
1535	pEXC-ΔBT3332_5r	catcctttttcttgtctggttggat	
1847	pEXC-ΔBT3332_3f	accagacaagaaaaaggatgaaagcattaaaaataacaatcatagctctattggca	
1848	pEXC-ΔBT3332_3r	aagataacattcgagtcgacatagaagctggctctttcgaaatagtc	
11492	pEXC-ΔBT1754_5f	cgggatccgtggactacttttgctgaaagcgga	engineer a chromosomal deletion of *BT1754*
11493	pEXC-ΔBT1754_5r	tccccccgggttcatagttctttctgtaatccaattaaga	
11494	pEXC-ΔBT1754_3f	tccccccgggtttcattgatatcgtaaagagggat	
11495	pEXC-ΔBT1754_3r	acgcgtcgactgccacacttccgtgcactt	
1880	pEXC-ΔBT1763_5f	gctctagaactagtggatcccagtaatgaagagacattacgg	engineer a chromosomal deletion of *BT1763*
1819	pEXC-ΔBT1763_5r	tagtttaatgttattaatttaaaagtacgaattttctcttttcgatg	
1839	pEXC-ΔBT1763_3f	aaattaataacattaaactaatgaaaaagataatatatatagcaacaatcggaattacc	
1840	pEXC-ΔBT1763_3r	aagataacattcgagtcgacctgtttcaggtcttcttcgttgattcc	

1972	pEXC-ΔBT1760_5f	gctctagaactagtggatccgcttcttccgtcagtcttct	engineer a chromosomal deletion of *BT1760*
1973	pEXC-ΔBT1760_5r	ttatttacacaagtagttgattgcattgagag	
1974	pEXC-ΔBT1760_3f	tcaactacttgtgtaaataatgaaaactacaccggcaagtaacatc	
1975	pEXC-ΔBT1760_3r	aagataacattcgagtcgactatcgcaacggggcggtgt	
2011	pEXC-ΔBT1759_5f	gctctagaactagtggatccctggaagatttgaaagcaactac	engineer a chromosomal deletion of *BT1759*
2012	pEXC-ΔBT1759_5r	tcaataagtgcttacctgaacgtctg	
2013	pEXC-ΔBT1759_3f	ttcaggtaagcacttattgaaaaacgactttcttctccctgc	
2014	pEXC-ΔBT1759_3r	aagataacattcgagtcgactgctccccacatggcaatgt	
2015	pEXC-ΔBT1760-59_3f	tcaactacttgtgtaaataaaaaacgactttcttctccctgc	engineer a chromosomal deletion of *BT1760-59*
2052	pEXC-ΔBT3082_5f	gctctagaactagtggatccccctctcaattggcgaaagaaaatc	engineer a chromosomal deletion of *BT3082*
2053	pEXC-ΔBT3082_5r	agctattttatttattagtttgtaaaatcggagt	
2054	pEXC-ΔBT3082_3f	cgattttacaaactaataaataaaatagctacggaaatcaaaagctatctttgttttcag	
2055	pEXC-ΔBT3082_3r	aagataacattcgagtcgacttccactggtaggctcgatg	
2058	pKO-BT1765_f	gctctagaactagtggatcctgggaacatttggctcctgc	engineer a chromosomal knock-out of *BT1765*
2059	pKO-BT1765_r	ggcccccccctcgaggtcgacatcattgtcctgtgtatagagtccc	
2077	pKO-BT1758_f	cgctctagaactagtggatccagaaaaccgtgttactcagtttgatcg	engineer a chromosomal knock-out of *BT1758*
2078	pKO-BT1758_r	gggcccccccctcgaggtcgacaaataacagagaacacattcgagttacc	
1978	pSIE1_ΔBACOVA_04496_5f	gattagcattatgaggatcctttggctatcccggcatcga	engineer a chromosomal deletion of *BACOVA_04496*
1979	ΔBACOVA_04496_5r	tgacgtgaatagttttgatttctcattttttcgtatttctttctatgacc	
1980	ΔBACOVA_04496_3f	aatcaaaactattcacgtcagaatacaataaatc	
1981	ΔSIE1_dBACOVA_04496_3r	tccaccgcggtggcggccgcgcagtatatacaaatagggttacgtct	
1791	pEXC-ΔBT2826_5f	gctctagaactagtggatcctgccgacagagatggtttaact	engineer a chromosomal deletion of *BT2826*
1792	pEXC-ΔBT2826_5r	aaatagccacataatactgtacaaaggtg	
1793	pEXC-ΔBT2826_3f	acagtattatgtggctatttatcgcattgataacttggacttagg	
1794	pEXC-ΔBT2826_3r	aagataacattcgagtcgaccatactcaaacatcttggaaatgaagcag	
**BT1760 complementation**			
1818	pNBU2-pBT1763_f	gctctagaactagtggatcctatcattcagttttctgttggttactttgag	Complement the *BT1760* deletion strain *in trans*
2007	P-BT1763-BT1760_f	cgtacttttaaattaataacattaaactaatgatgaaaaatatgatcttacctatagcat	
2008	pNBU2-BT1760_r	aagataggcaattagtcgactcaataagtgcttacctgaacgtc	
**SGBP over-expression**			
1723	pT7-7-H6-BT1761_f	agaaggagatatacatatgcatcaccatcaccatcacagtgatgacttcaaatccggcc	BT1761 overexpres

1724	pT7-7-BT1761_r	gcttatcatcgataagcttttatttacacaagtagttgattgcattgagag	sion in BL21 (DE3)
2087	pT7-6H-4G-BT3330_f	agaaggagatatacatatgcatcaccatcaccatcacggaggtggaggtgacgggctggacgaagcggtaggt	BT3330 overexpression in BL21 (DE3)
2088	pT7-7-BT3330_r	agcttatcatcgataagcttattccactacgtttaccaccat	

Supplementary Table 3. Plasmids used in this study.

Name	Description	Reference
pLYL01	empty multi-copy vector	[5]
pNBU2-ermG	empty single-copy vector conferring erythromycin resistance	[3]
pNBU2-tetQ	empty single-copy vector conferring tetracycline resistance	[3]
pNBU2-*lux-Pl*	The *Psuedorhabdus luminescens lux* cassette cloned into pNBU2-tetR	this work
pNBU2-*lux-Bt*	The *Bacteroides* optimized *lux* cassette cloned into pNBU2-tetR	this work
p-*lux-Pl*	The *Psuedorhabdus luminescens lux* cassette sub-cloned into pLYL01	this work
p-*lux-Bt*	The *Bacteroides* optimized *lux* cassette sub-cloned into pLYL01	this work
p*Bolux*	pLYL01 haboring BamHI and SpeI sites upstream of the *Bacteroides* optimized *lux* cassette	this work
P-*Bt-rpoD*	277 bp upstream of *BT1311* were cloned into the BamHI and SpeI sites in p*Bolux*	this work
P-*Bo-rpoD*	278 bp upstream of *BACOVA_00615* were cloned into the BamHI and SpeI sites in p*Bolux*	this work
P-*BT3332*	300 bp upstream of *BT3332* were cloned into p*Bolux*	this work
P-*BT1763*	300 bp upstream of *BT1763* were cloned into p*Bolux*	this work
P-*BACOVA_04505*	300 bp upstream of *BACOVA_04505* were cloned into p*Bolux*	this work
P-*BT2818*	300 bp upstream of *BT2818* were cloned into p*Bolux*	this work
pEXCHANGE-tdk	empty plasmid used to generate chromosomal deletions in *Bt*	[3]
pEXCHANGE-ΔBT3332	plasmid used to generate a chromosomal deletion of *BT3332*	this work
pEXCHANGE-ΔBT1763	plasmid used to generate a chromosomal deletion of *BT1763*	this work
pEXCHANGE-ΔBT1760	plasmid used to generate a chromosomal deletion of *BT1760*	this work
pEXCHANGE-ΔBT1759	plasmid used to generate a chromosomal deletion of *BT1759*	this work
pEXCHANGE-ΔBT1760-59	plasmid used to generate a chromosomal deletion of *BT1760-59*	this work
pEXCHANGE-ΔBT1754	plasmid used to generate a chromosomal deletion of *BT1754*	this work
pEXCHANGE-ΔBT3082	plasmid used to generate a chromosomal deletion of *BT3082*	this work
pKNOCK-ermG	plasmid to generate chromosomal insertions in *Bt*	[3]
pKNOCK-ermG-BT1765KO	plasmid used to inactivate *BT1765*	this work
pKNOCK-ermG-BT1758KO	plasmid used to inactivate *BT1758*	this work
pKNOCK-ermG-BT4410KO	plasmid used to inactivate *BT4410*	this work
pSIE1	empty plasmid to generate chromosomal deletions in *Bo*	[6]
pSIE1-ΔBACOVA_04496	plasmid to delete *BACOVA_04496* in *Bo*	this work
pT7-7	empty vector for protein over-expression	[7]
pT7-7-H6-4G-BT3330	pT7-7 construct for over-expression of BT3330 [D21-E347] with an N-terminal hexahistidine tag	this work
pT7-7-H6-BT1761	pT7-7 construct for over-expression of BT1761 [S25-K461] with an N-terminal hexahistidine tag	this work

Supplementary References

1. Cho KH, Salyers AA. Biochemical analysis of interactions between outer membrane proteins that contribute to starch utilization by Bacteroides thetaiotaomicron. *Journal of Bacteriology* **183**, 7224-7230 (2001).

2. Wood WB. Host specificity of DNA produced by Escherichia coli: bacterial mutations affecting the restriction and modification of DNA. *J Mol Biol* **16**, 118-133 (1966).

3. Koropatkin NM, Martens EC, Gordon JI, Smith TJ. Starch catabolism by a prominent human gut symbiont is directed by the recognition of amylose helices. *Structure* **16**, 1105-1115 (2008).

4. Raghavan V, Lowe EC, Townsend GE, 2nd, Bolam DN, Groisman EA. Tuning transcription of nutrient utilization genes to catabolic rate promotes growth in a gut bacterium. *Mol Microbiol* **93**, 1010-1025 (2014).

5. Reeves AR, D'Elia JN, Frias J, Salyers AA. A Bacteroides thetaiotaomicron outer membrane protein that is essential for utilization of maltooligosaccharides and starch. *J Bacteriol* **178**, 823-830 (1996).

6. Bencivenga-Barry NA, Lim B, Herrera CM, Trent MS, Goodman AL. Genetic Manipulation of Wild Human Gut Bacteroides. *J Bacteriol* **202**, (2020).

7. Tabor S, Richardson CC. A bacteriophage T7 RNA polymerase/promoter system for controlled exclusive expression of specific genes. *Proc Natl Acad Sci U S A* **82**, 1074-1078 (1985).
